# Multi-Omics Research Reveals the Effects of the ABA-Regulated Phenylpropanoid Biosynthesis Pathway on the UV-B Response in *Rhododendron chrysanthum* Pall.

**DOI:** 10.3390/plants14010101

**Published:** 2025-01-01

**Authors:** Wang Yu, Xiangru Zhou, Jinhao Meng, Xiaofu Zhou, Hongwei Xu

**Affiliations:** Jilin Provincial Key Laboratory of Plant Resource Science and Green Production, Jilin Normal University, Siping 136000, China; 15043512434@163.com (W.Y.); 17604342645@163.com (X.Z.);

**Keywords:** UV-B, *R. chrysanthum*, phenylpropanoid biosynthesis pathway

## Abstract

The growing depletion of the ozone layer has led to increased ultraviolet B (UV-B) radiation, prompting plants like the alpine *Rhododendron chrysanthum* Pall. (*R. chrysanthum*) to adapt to these harsh conditions. This study explored how abscisic acid (ABA) signaling influences *R. chrysanthum*’s metabolic responses under UV-B stress. *R. chrysanthum* was treated with UV-B radiation and exogenous ABA for widely targeted metabolomics, transcriptomics, and proteomics assays, and relevant chlorophyll fluorescence parameters were also determined. It was observed that UV-B stress negatively impacts the plant’s photosynthetic machinery, disrupting multiple metabolic processes. Multi-omics analysis revealed that ABA application mitigates the detrimental effects of UV-B on photosynthesis and bolsters the plant’s antioxidant defenses. Additionally, both UV-B exposure and ABA treatment significantly influenced the phenylpropanoid biosynthesis pathway, activating key enzyme genes, such as 4CL, CCR, and HCT. The study also highlighted the MYB–bHLH–WD40 (MBW) complex’s role in regulating this pathway and its interaction with ABA signaling components. These findings underscore ABA’s crucial function in improving plant resistance to UV-B stress and offer novel insights into plant stress biology.

## 1. Introduction

In recent years, substantial degradation of the ozone layer due to increasing environmental pollution has led to excessive ultraviolet B (UV-B) radiation (280 to 315 nm) exposure on the earth’s surface [1]. Excess UV-B radiation has substantial adverse effects on plants [2]. UV-B stress is currently demonstrated to produce reduced leaf area and even defoliation in plants [3]. UV-B stress can also disrupt photosynthesis in plants by degrading plant Photosystem II (PSII) proteins, impairing PS electron donors, and even interfering with stomatal function, resulting in a decrease in photosynthesis rates [4,5,6]. UV-B stress causes the production of reactive oxygen species (ROS) in plants, and excessive accumulation of these substances disrupts various metabolic processes in plant cells [7,8]. UV-B stress also alters plant metabolite content, causing significant changes in the total sugar and starch content, as well as a significant increase in carotenoid and phenolic levels [9,10,11]. As a result, in response to the environmental changes caused by the ever-increasing UV-B radiation, various plants have gradually developed their own distinct adaptive mechanisms to cope with UV-B stress.

*Rhododendron chrysanthum* Pall. (*R. chrysanthum*), a perennial evergreen dwarf shrub, mainly grows in the high altitudes of Changbai Mountains [12]. Because of the harsh environmental conditions in the Changbai Mountains, *R. chrysanthum* has evolved resistance to strong UV radiation, low temperatures, and other abiotic stresses. Previous research on *R. chrysanthum* revealed that UV-B stress causes reprogramming of its major primary metabolism pathways [13]. *R. chrysanthum*’s PSII proteins undergo acetylation alteration in response to UV-B stress, and this modification partially offsets the plant’s UV-B damage [14]. However, few studies have been conducted on the phenylpropanoid biosynthesis pathway in the reaction to UV-B stress in *R. chrysanthum*, which is ubiquitous and plays a vital function in plants and hence warrants more investigation.

The phenylpropanoid biosynthesis pathway acts as a crucial part in plant metabolism. This pathway is capable of efficiently producing a diverse range of important secondary metabolites that play critical roles in plant reactions to varied environmental stressors [15,16]. The phenylpropanoid biosynthesis pathway is a major source of most defensive secondary metabolites (flavonoids, lignans, salicylic acid, etc.) and is capable of efficiently scavenging reactive oxygen species accumulated as a result of various stresses, as well as playing an important regulatory role in the reorientation of metabolic streams in different pathways [17]. Among these metabolites, flavonoids, as an important class of derivatives in the phenylpropanoid biosynthesis pathway, are able to absorb UV-B rays at 280–315 nm so that plant organs and tissues, especially photosynthetic tissues, can be protected from UV-B damage, and are important protective barriers against the absorption of ultraviolet radiation in plants [18]. Furthermore, flavonoids have strong antioxidant activity, which can scavenge reactive oxygen species produced during stress and increase plant survival [19]. Therefore, the study of the dynamics of the phenylpropanoid biosynthesis pathway under abiotic stress in *R. chrysanthum* is of great importance.

Abscisic acid (ABA), an abiotic stress hormone that increases plant resistance, serves as both a signal for the plant response to abiotic stress and an important factor in eliciting adaptive regulatory responses and gene expression in plants [20]. Plants are able to regulate the degree of stomatal opening and closing through ABA to reduce the plant transpiration rate in order to counteract the damage caused by abiotic stresses on plants [21]. ABA can also activate the antioxidant defense system, which scavenges reactive oxygen species produced by environmental stress [22]. Numerous experiments on exogenous ABA have shown that exogenous ABA can significantly enhance the stress tolerance performance of plants [23,24,25]. Few studies have been carried out on the synergistic responses of the phenylpropanoid biosynthesis pathway in plants to UV-B stress that are regulated by ABA, despite the fact that the function of this pathway in plants cannot be overlooked and that ABA is crucial for improving plant stress tolerance.

The benefits of both targeted and untargeted metabolomics are combined in widely targeted metabolomics analysis, which has the ability to accurately respond to the dynamic changes in plant metabolites. Its features include high throughput, ultra-sensitivity, wide coverage, and accurate qualitative and quantitative analysis [26,27]. The transcriptome can accurately reflect changes in the expression of various genes in plants as a result of environmental changes, aiding in the discovery of regulatory relationships between genes and networks [28]. Protein acetylation, a widespread post-translational modification on lysine, tweaks protein properties, influencing activity and cell signaling [29,30]. Protein acetylation modificationomics is precisely the most important research tool to reveal protein acetylation, providing new perspectives for understanding life processes [31].

Given the crucial role of the abscisic acid and phenylpropanoid biosynthesis pathways in plant reactions to adversity stress, this study investigated the molecular mechanisms governing how ABA regulates the phenylpropanoid biosynthesis route in *R. chrysanthum* in response to UV-B radiation. The dynamic alterations in *R. chrysanthum* ABA biosynthesis and signaling under UV-B stress were investigated further via a joint multi-strategyomics analysis.

## 2. Results

### 2.1. R. chrysanthum’s Photosynthetic System Is Injured by UV-B Exposure

In order to demonstrate the detrimental impact of UV-B stress on *R. chrysanthum*’s photosynthetic system, radargrams illustrating the pertinent OJIP (fast chlorophyll a fluorescence induction curve) test parameters were created (Supplementary Figure S1A). The findings indicated that following exposure to UV-B radiation, the levels of φ*D*_o_ (quantum yield at t = Fo for energy dissipation), Fo (minimal fluorescence), and S_m_ (normalised total complementary area) rise, whereas *Ψ*_o_ (probability that a trapped exciton moves an electron into the electron), φ*E*_o_ (quantum yield at t = Fo for electron transport), and Fm (fluorescence maximum) all fall. UV-B radiation also resulted in a significant decrease in PI_ABS_ (performance index on absorption basis) and Fv/Fm (maximal photochemical efficiency of PSII) and a significant increase in DIo/RC (dissipated energy flux per RC at t = Fo) in *R. chrysanthum* (Supplementary Figure S1B–D).

### 2.2. Response of Key Metabolites in R. chrysanthum to UV-B Radiation

**Figure 1:**
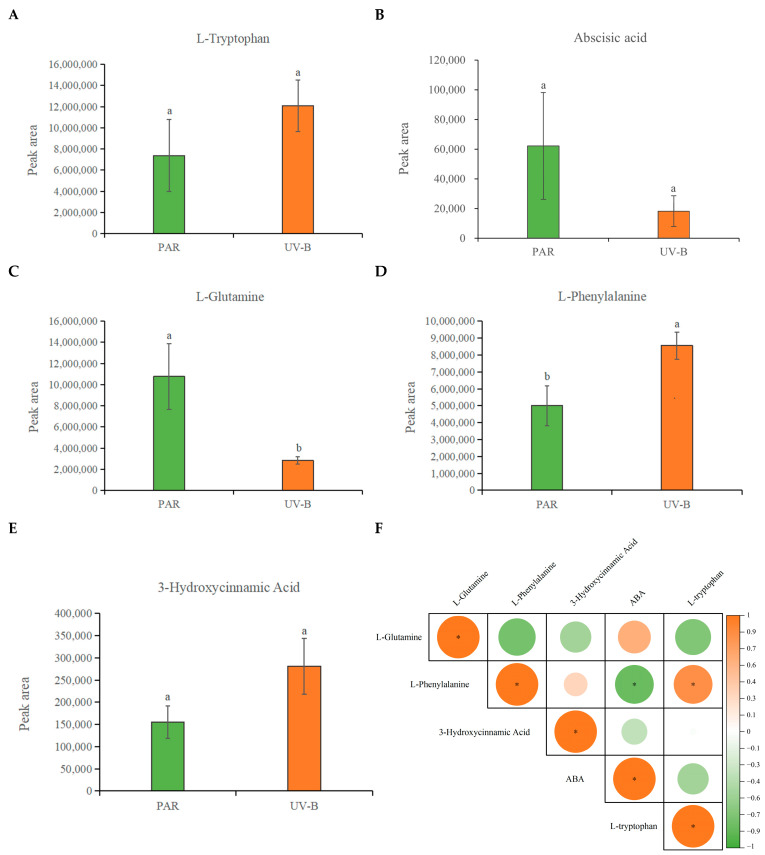

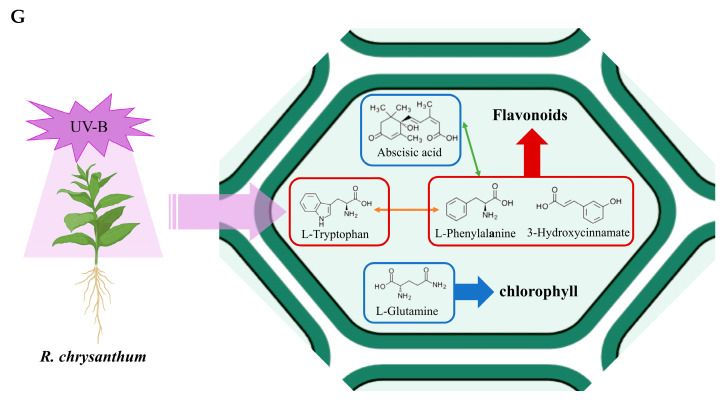
After UV-B radiation exposure, *R. chrysanthum*’s ABA and key metabolites act together. (**A**–**E**) Bar graphs representing the changes in L-tryptophan, ABA, L-glutamine, L-phenylalanine, and 3-hydroxycinnamic acid after UV-B radiation exposure, respectively. The peak area is used to estimate the relative amount of a specific metabolite in a sample. The larger the peak area, the higher the amount of that metabolite in the sample. The three biological duplicate experiments’ means are shown by the heights of the bar graphs (*n* = 3), and the SDs of the three samples are shown by the error bars. Significant changes across data groups are indicated by different letter markers (*p* < 0.05). (**F**) Correlation analysis between key substances in *R. chrysanthum* under UV-B radiation. Correlation analyses were performed on the data involved for each metabolite in (**A**–**E**). Orange and green circles represent positive and negative correlations, respectively, and the sizes and color shades of the circles indicate the strength of the correlation, with “*” representing r^2^ ≥ 0.8 and *p* < 0.05. (**G**) Mechanogram of changes in ABA and key metabolites in *R. chrysanthum* after UV-B radiation exposure. Orange bi-directional arrows represent positive correlations, and green bi-directional arrows represent negative correlations.

UV-B radiation resulted in an increase in L-tryptophan content and a decrease in ABA content in *R. chrysanthum*, and the differences in these changes were not significant (Figure 1A,B). L-tryptophan is able to participate in the signaling process of plants in response to UV-B stress, and the increase in its content due to UV-B radiation indicates that *R. chrysanthum* switched on a response mechanism upon exposure to UV-B radiation. L-glutamine is associated with photosynthetic pigments, and UV-B caused a decrease in its content, suggesting that UV-B can have some effect on the photosynthetic system of *R. chrysanthum*, which is consistent with the aforementioned results, indicating that UV-B can disrupt the photosynthetic system of *R. chrysanthum* (Figure 1C).

The biosynthetic process of phenylpropanoid involves critical roles of L-phenylalanine and 3-hydroxycinnamic acid. The results of the study showed a significant increase in L-phenylalanine content due to UV-B radiation, and 3-hydroxycinnamic acid also increased but not significantly (Figure 1D,E). This occurrence reveals that the phenylpropanoid biosynthesis pathway may be activated in *R. chrysanthum* in its reaction to UV-B exposure.

Correlation analyses showed that the aforementioned key metabolites in *R. chrysanthum* were closely linked to each other under UV-B radiation, among which L-phenylalanine was more strongly correlated with L-tryptophan and ABA (Figure 1F). Consequently, through dynamic alterations in ABA and phenylalanine biosynthesis, *R. chrysanthum* may react favorably to UV-B stress (Figure 1G).

### 2.3. Treatment with Exogenous ABA Protects R. chrysanthum’s Photosynthetic System

**Figure 2:**
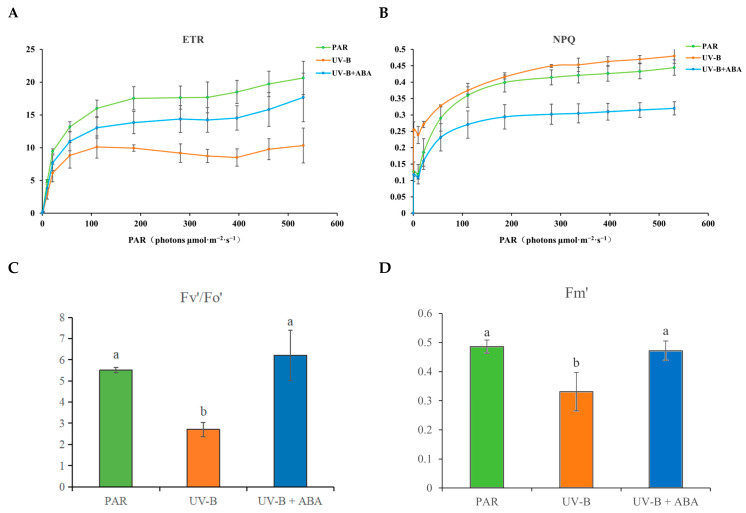
Dynamics of *R. chrysanthum*’s chlorophyll fluorescence characteristics. (**A**,**B**) Line plots of NPQ and ETR in *R. chrysanthum* after exogenous ABA and UV-B radiation treatments. (**C**,**D**) Histograms of Fv′/Fo′ (variable fluorescence ratio in the light) and Fm′ (maximum fluorescence in the light) of PSII of *R. chrysanthum* after treatment with exogenous ABA and UV-B radiation. The three biological duplicate experiments’ means are shown by the heights of the bar graphs (*n* = 3), and the SDs of the three samples are shown by the error bars. Significant changes across data groups are indicated by different letter markers (*p* < 0.05).

This study revealed that while UV-B exposure decreased *R. chrysanthum*’s actual electron transport rate (ETR), exogenous ABA prevented this downward trend, hence minimizing the effect (Figure 2A). Non-photochemical quenching (NPQ) is a protective mechanism for plants to avoid damage to the photosynthetic apparatus caused by excess excitation energy produced during photosynthesis. The rise in NPQ after UV-B radiation exposure suggests that *R. chrysanthum* protects the photosynthetic apparatus from damage by UV-B radiation by enhancing energy dissipation. In contrast, the decrease in NPQ after exogenous ABA treatment suggests that ABA may disperse too much excess excitation energy by regulating the phenylpropanoid biosynthesis pathway, thus helping *R. chrysanthum* to respond positively to UV-B radiation (Figure 2B).

All of the assessed chlorophyll fluorescence parameters dramatically decreased, having been exposed to UV-B radiation; nonetheless, exogenous ABA significantly reversed this trend and increased *R. chrysanthum*’s potential to withstand stress (Figure 2C,D). All things considered, the findings demonstrate that *R. chrysanthum*’s photosynthetic system is negatively impacted by UV-B radiation and that exogenous ABA could improve *R. chrysanthum*’s resistance to stress.

### 2.4. Alteration of the Phenylpropanoid Biosynthesis Pathway and the Derivative Flavonoids of This Pathway in R. chrysanthum

**Figure 3:**
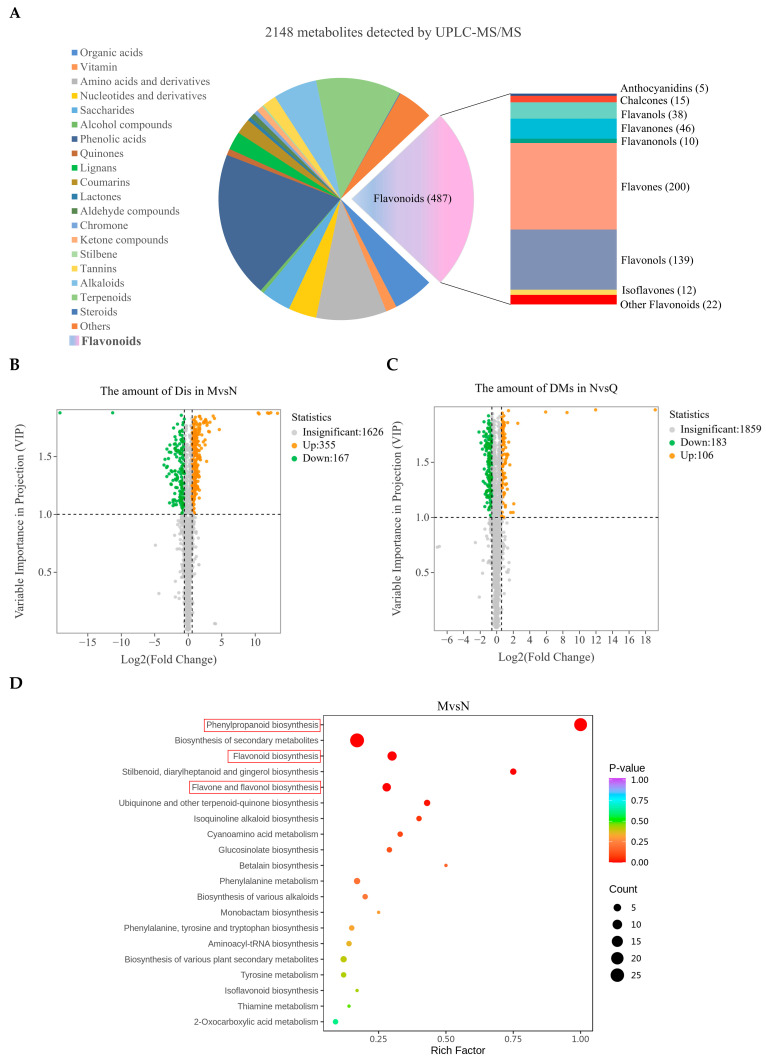

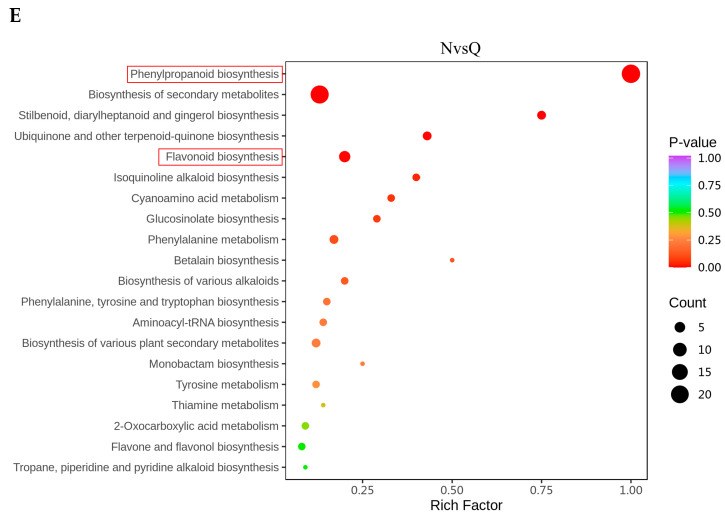
Information related to metabolites in *R. chrysanthum* detected by widely targeted metabolomics. Group M (PAR treated), group N (UV-B radiation treated), and group Q (exogenous ABA and UV-B treated) were the three groups being studied. (**A**) Classification statistics of detected flavonoids and their percentage in total metabolites. (**B**) Volcano plots of DM counts in *R. chrysanthum* under UV-B radiation. (**C**) Volcano plots of DM counts in *R. chrysanthum* after exogenous ABA treatment. (**D**) Bubble plots of KEGG enrichment analysis of DMs in *R. chrysanthum* under UV-B radiation. (**E**) Bubble plots of KEGG enrichment analysis of DMs in *R. chrysanthum* after exogenous ABA treatment.

The findings from widely targeted metabolomics revealed 2148 *R. chrysanthum* metabolites in total. With 487 species, the majority of them were flavonoids and could be divided into nine main families (Figure 3A). Different metabolites (DMs) and different flavonoids (DFs) were screened according to variable importance in the projection (VIP) and fold change (FC) values. UV-B radiation (MvsN) resulted in 522 DMs (355 raised and 167 reduced) in *R. chrysanthum*, while exogenous ABA treatment (NvsQ) resulted in 289 DMs (183 raised and 106 reduced) (Figure 3B,C). KEGG enrichment analyses showed that post-UV-B, the DMs of *R. chrysanthum* were mainly enriched in phenylpropanoid biosynthesis, flavonoid biosynthesis, and flavone and flavonol biosynthesis, and the DMs after exogenous ABA treatment were similarly mainly concentrated in the phenylpropanoid biosynthesis and flavonoid biosynthesis (Figure 3D,E). This suggests that UV-B radiation activates phenylpropanoid biosynthesis in *R. chrysanthum*, while exogenous ABA modulates the phenylpropanoid biosynthesis pathway, thereby inducing a positive response to UV-B in *R. chrysanthum*.

### 2.5. Two Experimental Treatments Cause Reprogramming of the Phenylpropanoid Biosynthesis Pathway (ko00940) in R. chrysanthum

This experiment was conducted on *R. chrysanthum* leaf samples for transcriptomics to investigate the dynamics of genes involved in the phenylpropanoid biosynthesis pathway and its internal ABA biosynthesis, as well as the response of genes related to the signaling pathway to UV-B. KEGG enrichment analyses of DEGs produced by the two treatments showed that UV-B radiation mainly led to the production of 35 differentially expressed genes (DEGs) in the phenylpropanoid biosynthesis of *R. chrysanthum*, and exogenous ABA treatment resulted in the production of 3 DEGs (Supplementary Figure S2).

**Figure 4:**
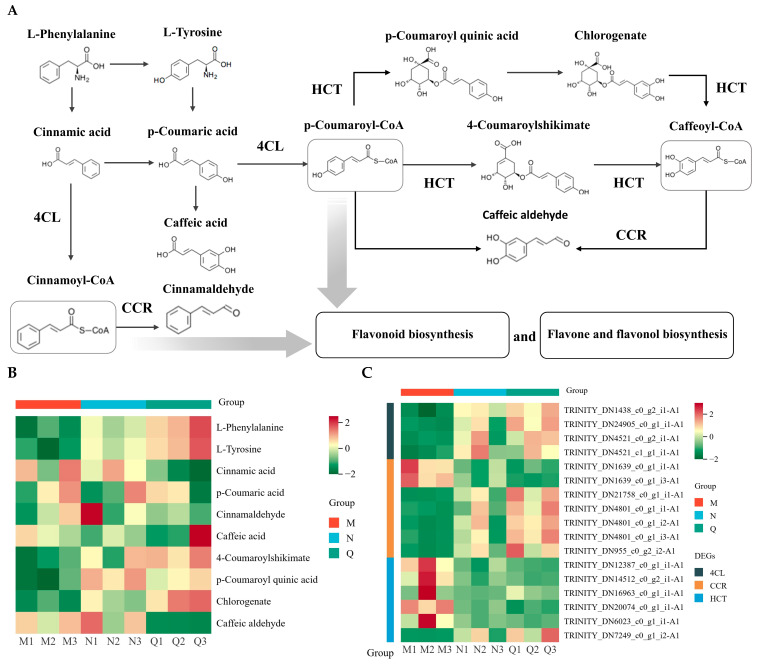
Two experimental treatments prompted reprogramming of the phenylpropanoid biosynthesis pathway in *R. chrysanthum*. Group M (PAR treated), group N (UV-B radiation treated), and group Q (exogenous ABA and UV-B treated) were the three groups being studied. (**A**) Simplified modeling of the phenylpropanoid biosynthesis pathway in *R. chrysanthum*. (**B**,**C**) Clustering heatmap of DMs and DEGs. The expression of DMs and DEGs involved in enzymes in the pathway is presented as a clustered heatmap; the higher the expression, the more reddish the color, and the lower the expression, the more greenish the color. From left to right, each of the adjacent three squares represents three biological replicates of each of the three comparison groups (M, N, and Q).

The simplified model of the phenylpropanoid biosynthesis pathway illustrated alterations in DMs and DEGs in phenylpropanoid biosynthesis in *R. chrysanthum* following the two experimental treatments, as well as the regulatory link between them (Figure 4A). ABA and UV-B led to the differential expression of a large number of metabolites in the phenylpropanoid biosynthesis pathway. Among them, the expression of most DMs increased under the influence of UV-B and further increased after ABA treatment, such as the initiating metabolites L-phenylalanine and L-tyrosine (Figure 4B). Following the two experimental treatments, DEGs encoding three enzymes involved in the pathway of phenylpropanoid biosynthesis were generated. There were four DEGs involved in encoding 4-coumarate-CoA ligase (4CL, [EC:6.2.1.12]), and two DEGs were up-regulated in MvsN and consistently up-regulated in NvsQ. There were seven DEGs involved in encoding cinnamoyl-CoA reductase (CCR, [EC:1.2.1.44]), five DEGs were up-regulated in MvsN and consistently up-regulated in NvsQ, and two DEGs were down-regulated in MvsN and consistently down-regulated in NvsQ. There were six DEGs involved in encoding shikimate. There were six DEGs involved in encoding shikimate O-hydroxycinnamoyltransferase (HCT, [EC:2.3.1.133]), of which one DEG was up-regulated in MvsN and consistently up-regulated in NvsQ, and four DEGs were down-regulated in MvsN and consistently down-regulated in NvsQ (Figure 4C).

The findings reveal that the phenylpropanoid biosynthesis pathway is activated in the presence of UV-B exposure in *R. chrysanthum* and exogenous ABA plays an important role in the regulation of enzymes and metabolites related to the phenylpropanoid biosynthesis pathway. This phenomenon is also consistent with the results of metabolomics KEGG analyses, suggesting that UV-B radiation and exogenous ABA mainly affect the phenylpropanoid biosynthesis pathway in *R. chrysanthum*. This phenomenon is also consistent with the results of metabolomics KEGG analysis, suggesting that the two experimental treatments mainly affected the phenylpropanoid biosynthesis pathway in *R. chrysanthum*.

### 2.6. MBW Complex Is Involved in the Transcriptional Regulation of the Phenylpropanoid Biosynthesis Pathway in R. chrysanthum

**Figure 5:**
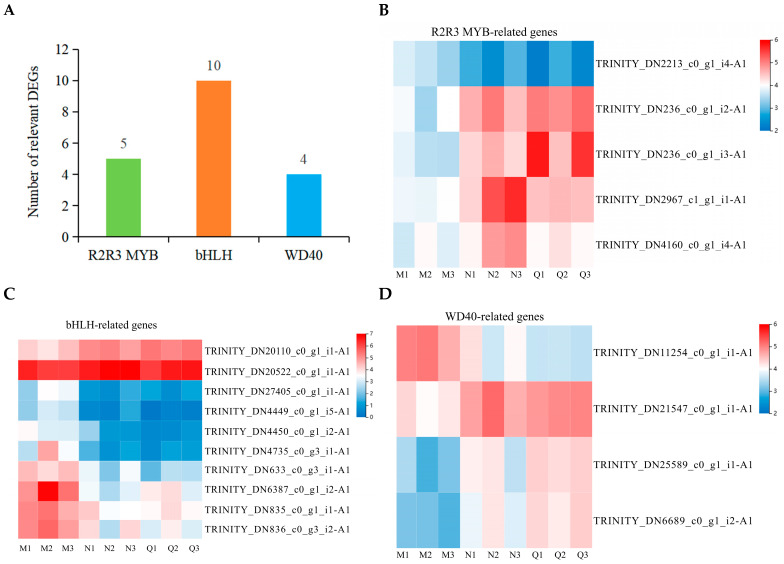
Dynamics of the MBW complex after two experimental treatments. (**A**) Quantitative statistics of MYB, bHLH, and WD40-associated DEGs in *R. chrysanthum* after two experimental treatments. (**B**–**D**) The heatmap of clustering of the expression of MYB, bHLH, and WD40-related DEGs.

The phenylpropanoid biosynthesis pathway is regulated by a variety of signaling pathways and multiple regulatory mechanisms, among which the MBW complex, consisting of transcription factors and regulatory proteins (R2R3-MYB, bHLH, and WD40), plays an important role in regulating the phenylpropanoid biosynthesis pathway. In this study, we found that a total of 5 R2R3-MYB, 10 bHLH, and 4 WD40-related genes were differentially accumulated in *R. chrysanthum* after two experimental treatments (UV-B and exogenous ABA treatments) (Figure 5A). Most bHLH-related genes were down-regulated after UV-B radiation exposure and consistently down-regulated after exogenous ABA treatment, and only one related gene was consistently elevated after both treatments. In contrast, most WD40-related genes were up-regulated after UV-B radiation exposure and persistently up-regulated after exogenous ABA treatment. Two of the MYB-related genes were consistently up-regulated and one consistently down-regulated after both treatments (Figure 5B–D) (Table 1).

Correlation analyses of DMs and DEGs in the phenylpropanoid biosynthesis pathway in *R. chrysanthum* were performed to explore the link between the MBW complex and phenylpropanoid biosynthesis (Supplementary Figure S3). The results showed that the MBW complex was significantly associated with genes related to 4CL, CCR, and HCT. In addition, the MBW complex was significantly correlated with L-phenylalanine and L-tyrosine, the initiating metabolites of the phenylpropanoid biosynthesis pathway, suggesting that there is a close association between the MBW complex of *R. chrysanthum* and the phenylpropanoid biosynthesis pathway under both experimental treatments.

### 2.7. ABA Production and Signaling in R. chrysanthum Regulate the Phenylpropanoid Biosynthesis Pathway in the Aftermath of UV-B Stress

In addition to the phenylpropanoid biosynthesis pathway being regulated by transcription factors, phytohormone-signaling-related pathways play an important role. The phenylpropanoid biosynthesis pathway in *R. chrysanthum* is, indeed, regulated by ABA, as demonstrated by the preceding exogenous ABA-related data. In this experiment, we further investigated the biosynthesis process of ABA in *R. chrysanthum* under UV-B radiation and its alterations in key genes associated with it as a signal during signal transduction in order to present a complete picture of the dynamics of ABA in regulating the phenylpropanoid biosynthesis pathway in response to UV-B stress.

**Figure 6:**
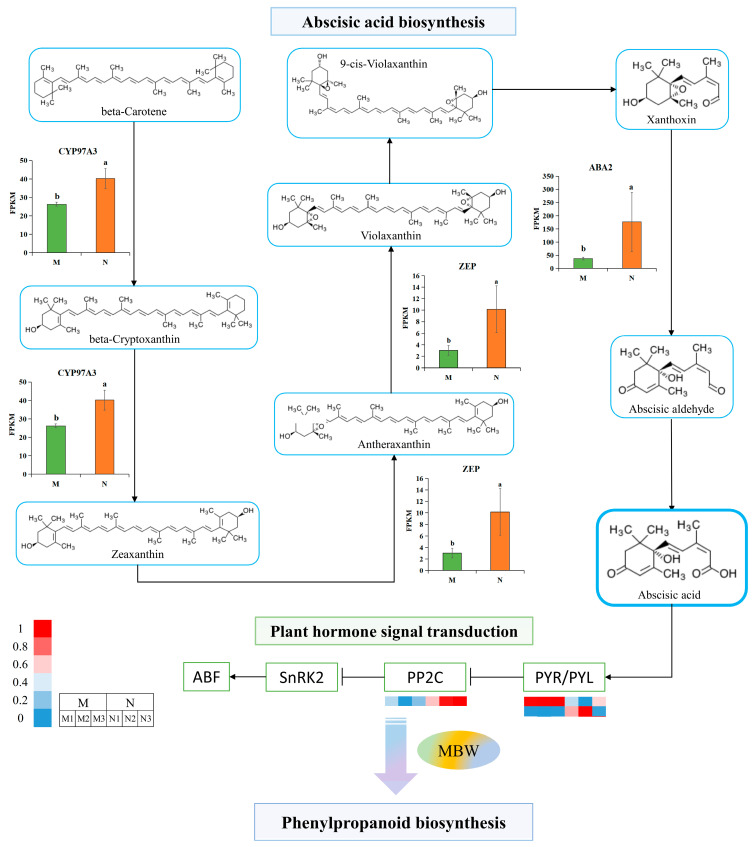
A simplified model of ABA biosynthesis and signal transduction in *R. chrysanthum*. The three biological duplicate experiments’ means are shown by the heights of the bar graphs (*n* = 3), and SDs of the three samples are shown by the error bars. Significant changes across data groups are indicated by different letter markers (*p* < 0.05). The expression of DEGs in the ABA signal transduction pathway is presented as a clustered heatmap; the higher the expression, the more reddish the color, and the lower the expression, the more bluish the color. From left to right, each of the adjacent three squares represents the respective three biological replicates of the two comparison groups, M and N, respectively.

The findings demonstrated that during *R. chrysanthum*’s ABA biosynthesis, UV-B raised the majority of the important enzymes (CYP97A3, ZEP, and ABA2). UV-B radiation caused differential accumulation of two PYL-related genes (one up-regulated and one down-regulated) and one PP2C-related gene during ABA signaling in *R. chrysanthum* (Figure 6, Table 2). It is thus evident that changes in these key ABA-related genes in *R. chrysanthum* turn on their own ABA signaling pathways to respond positively to UV-B stress.

### 2.8. Acetylation Modification of SnRK2, a Key Enzyme in the ABA Signal Transduction Pathway in R. chrysanthum, Under UV-B Stress

Protein acetylation modifications exert a significant impact on the activation of the signal transduction pathway, as well as the activity of signaling molecules. In this study, we performed acetylation proteomics of proteins related to the ABA signal transduction process in *R. chrysanthum* under UV-B radiation in order to reveal the effect of UV-B radiation on the related proteins in ABA signal transduction at the level of acetylation modification.

**Figure 7:**
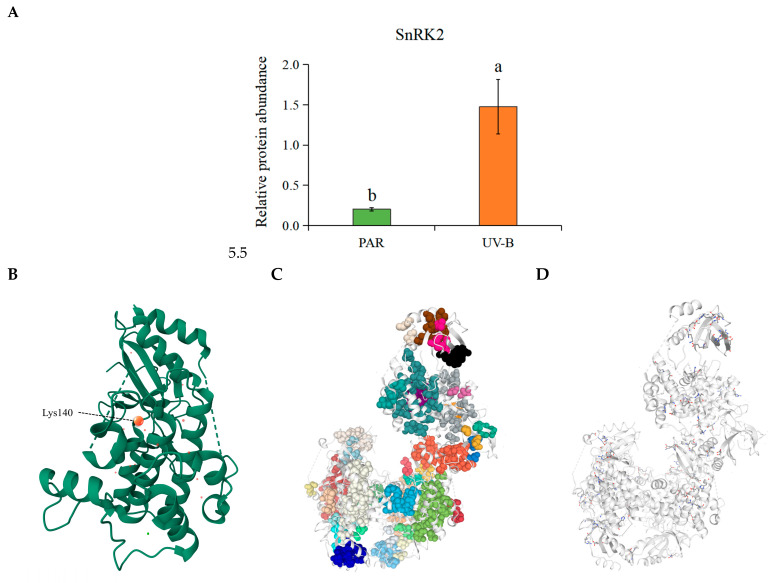
Information about SnRK2 in *R. chrysanthum* under UV-B stress. (**A**) Bar chart of SnRK2 expression under UV-B stress. The three biological duplicate experiments’ means are shown by the heights of the bar graphs (*n* = 3), and the SDs of the three samples are shown by the error bars. Significant changes across data groups are indicated by different letter markers (*p* < 0.05). (**B**) Visualization of the three-dimensional structure of SnRK2 and labeling of its acetylation sites. (**C**) Visualization of SnRK2 hydrophobic clusters. (**D**) Visualization of SnRK2 salt bridges.

The results showed that lysine residue 140 of the SnRK2 protein in the ABA signal transduction pathway in *R. chrysanthum* underwent significant acetylation modification, and its expression was significantly increased after UV-B radiation treatment (Figure 7A). Based on this, a three-dimensional structure of SnRK2 was constructed in this experiment, and the acetylation site was labeled (Figure 7B).

Serine/threonine-protein kinase SRK2 (SnRK2) is able to perform its corresponding biological functions, thanks to its natural structure, and the driving of various non-covalent interactions is crucial for the stabilization of its natural structure. Therefore, this experiment used ProteinTools to visualize and analyze these structures (Figure 7C,D). The results showed that SnRK2 contained 38 hydrophobic clusters, of which the largest hydrophobic cluster area was 4014.6^2^ (cluster 3), and the smallest hydrophobic cluster area was 38.12^2^ (cluster 14). Cluster 3 contained 28 residues, with an area of 39.75^2^ per residue and 101 interactions between residues, and cluster 14 contained 2 residues, with an area of 38.12^2^ per residue and 2 interactions between residues (Supplementary Table S1). In addition, 70 salt bridge networks were found to exist in the protein, and charge separation parameters were calculated. The findings revealed that the kappa value was 0.13 and the FCR was 0.26.

Accordingly, *R. chrysanthum* is likely to activate the downstream phenylpropanoid biosynthesis pathway under UV-B stress conditions, probably by regulating ABA biosynthesis and interacting with the ABA signaling pathway and ultimately activating the downstream phenylpropanoid biosynthesis pathway through the modification of the acetylation of SnRK2 proteins, which in turn triggers a series of positive responses.

## 3. Discussion

The ozone layer absorbs the majority of UV-B rays in solar radiation and serves as the earth’s primary barrier to UV radiation; hence, its loss has resulted in severe UV-B stress for all life on earth [32]. Plants cannot actively avoid hazards but can only face the environmental stresses they face through different specialized adaptive mechanisms [33]. Therefore, in the face of environmental stresses, plants regulate their key metabolic pathways to synthesize important secondary metabolites to ensure that they can survive [34,35].

Photosynthesis in plants converts solar energy into chemical energy and is a direct source of the materials and energy needed for growth and development [36]. Fo is the fluorescence intensity of the chlorophyll molecule in its most basal state, which rises when the plant is subjected to environmental stresses; *Ψ*_o_ represents the plant’s use of light energy and the efficiency of energy conversion during photosynthesis [37]. φ*D*_o_ can show the quantum ratio used for heat dissipation, S_m_ responds to the capacity of the electron acceptor reservoir on the acceptor side, and φ*Eo* indicates the quantum yield used for electron transfer [38]. This demonstrates that UV-B radiation degrades *R. chrysanthum*’s antenna pigments, and excess light energy is lost as heat, disrupting the normal operation of the photosynthetic electron transport chain and impeding photosynthesis (Supplementary Figure S1A). PI_ABS_ is an index of PSII performance based on absorbed light energy that provides an integrated response to the state of the photosynthetic apparatus, Fv/Fm is the maximum photochemical efficiency, and DIo/RC represents the degree of heat dissipation per reaction center [39,40]. The results of this study illustrate that UV-B radiation caused some damage to electron transfer in the PSII of *R. chrysanthum*, and the increase in the burden on the active reaction center forced an increase in the energy dissipation efficiency and heat dissipation (Supplementary Figure S1B–D).

The maximum absorption peak of L-tryptophan in solution is near 280 nm, so it is considered as one of the candidates for sensing UV-B chromophores [41]. L-glutamine is involved in the conversion process from amino acids to chlorophyll precursors and is one of the key nitrogen sources for chlorophyll biosynthesis [42,43]. L-phenylalanine is the initiating metabolite of the phenylpropanoid biosynthesis pathway, and the key precursor for flavonol synthesis is 3-hydroxyphenylpropionic acid, which consists of 3-hydroxycinnamic acid, able to pass through shikimate O-hydroxycinnamoyl transferase (HCT)-catalyzed synthesis [44,45]. The results of this study showed that these key metabolites were altered to different extents after UV-B radiation exposure, while correlation analyses indicated that ABA was likely involved in regulating the phenylpropanoid biosynthesis pathway in *R. chrysanthum* under UV-B stress (Figure 1).

It has been shown that oilseed rape is able to activate its own phenylpropanoid biosynthesis pathway and thus regulate the phenolic acid biosynthesis pathway in order to respond positively to drought stress [46]. Under salt stress, the soybean’s phenylpropanoid biosynthesis pathway is activated, which helps control flavonoid synthesis and prevent the formation of reactive oxygen species in the body [47]. The results of this study showed that UV-B radiation and exogenous ABA treatment mainly altered the phenylpropanoid biosynthesis pathway in *R. chrysanthum* (Figure 3B,C). Combined with the results of chlorophyll fluorescence parameters, it is likely that exogenous ABA affects flavonoid biosynthesis by regulating the phenylpropanoid biosynthesis pathway, thereby protecting the photosynthetic system of *R. chrysanthum* (Figure 2). In addition, metabolomics analyses showed that flavonoids accounted for the largest proportion of all metabolites detected in *R. chrysanthum*, which also coincided with a significant increase in 3-hydroxycinnamic acid after UV-B radiation exposure (Figure 1E and Figure 3A). The phenylpropane biosynthesis pathway relies on the regulation of a series of enzymes to ensure that the pathway proceeds properly [48]. It has been shown that the activation of the 4CL gene is closely related to plant defense mechanisms [49]. The results of this study are consistent with previous findings that UV-B radiation and exogenous ABA are able to differentially express 4CL-related genes in *R. chrysanthum* (Figure 4C). This is further evidence from a transcriptomic perspective that the phenylpropanoid biosynthesis pathway in *R. chrysanthum* responds to UV-B stress and that ABA plays an important role in regulating the phenylpropanoid biosynthesis pathway.

It has been shown that the MBW complex, composed of three regulatory proteins (MYB, bHLH, and WD40), is able to regulate the entire phenylpropane metabolic pathway in Arabidopsis [50]. Not only that, one of the R2R3-MYB transcription factors is also closely related to flavonoid regulation [51]. While the bHLH transcription factor does not actually alter flavonoid production pathways, it can dramatically regulate these pathways when combined with the MYB transcription factor [52]. WD40 transcription factors, also known as WDR proteins, play an important role in the regulation of flavonoid metabolism and are also able to assist plants in responding positively to the various stresses they face [53]. In this study, we found that exogenous ABA was able to further alter most of the relevant DEGs of the MBW complex in *R. chrysanthum* after UV-B radiation exposure in the same trend (Table 1). Correlation analyses showed that the MBW complex was strongly associated with key enzymes in the phenylpropanoid biosynthesis pathway (4CL, CCR, and HCT), as well as with key metabolites, such as L-phenylalanine (Supplementary Figure S3). These results suggest that the MBW complex, indeed, has an important effect on the phenylpropanoid biosynthesis pathway in *R. chrysanthum*.

It has been shown that the expression of genes, such as C4H, DFR, and LDOC, in Arabidopsis seedlings after treatment with exogenous ABA elevated the content of anthocyanins in *Arabidopsis thaliana* [54]. Plant ABA signaling can regulate ionic and osmotic homeostatic signaling-related pathways to maintain its own cellular homeostasis under salt stress [55]. In this study, we found that five key enzymes in ABA biosynthesis were differentially expressed after UV-B radiation exposure (CYP97A3, ZEP, and ABA2, as well as PYL and PP2C) in the ABA signaling pathway (Figure 6). This suggests that UV-B radiation induces changes in ABA biosynthesis and signal transduction in *R. chrysanthum* and thus regulates the downstream phenylpropanoid biosynthesis pathway.

Serine/threonine-protein kinase SRK2 (SnRK2) and PP2C are able to interact with each other, and in general, PP2C inhibits SnRK2 activity, which in turn silences ABA signaling [56]. In contrast, environmental stress can stimulate PYR/PYL to interact with and inhibit the activity of PP2C, leading to the release of SnRK2 from the negative regulatory system by PP2C and the conversion of signaling molecules by the phosphorylation of the downstream factors of SnRK2 and PP2C, which in turn has an impact on subsequent metabolic pathways [57]. This investigation found that PP2C-associated DEGs increased with UV-B, while SnRK2 itself underwent acetylation (Figure 6 and Figure 7). Although UV-B increased the expression of PP2C-related genes capable of inhibiting the activity of SnRK2, it also caused the acetylation of SnRK2. This twofold effect on SnRK2 mediated by UV-B radiation is most likely a unique response style of *R. chrysanthum* to UV-B, and the way it works warrants further exploration.

The results of this study are of great significance for the conservation of *R. chrysanthum* germplasm resources and obtaining new perspectives for understanding the changing patterns of metabolites and their regulatory mechanisms in plants under adverse conditions. The results highlight the key role of ABA in regulating the phenylpropanoid biosynthesis pathway in plants in response to UV-B stress, which broadens research in the field of plant adversity biology. In addition, a deeper understanding of the mechanisms of plant adaptation to UV-B radiation is crucial for the maintenance of biodiversity, especially in ecosystems that are extremely sensitive to changes in UV-B radiation. As global climate change continues, these findings have long-term implications for enhancing agricultural productivity.

## 4. Materials and Methods

### 4.1. The Cultivation and Radiation Treatment of Experimental Material

The experimental material in this study was *R. chrysanthum*. To ensure optimal growth of experimental materials, they were kept in an intelligent artificial climate room that simulated an alpine environment. Samples were cultured in 1/4 MS medium. The smart artificial climate chamber simulated the natural light–dark cycle (14 h light/10 h dark) and maintained appropriate temperature conditions (18 °C daytime/16 °C nighttime) and a photon flux density of 50 µmol (photon) $m^{-2}$ $s^{-1}$. The experiment was conducted on 8-month-old and similarly growing cultured seedlings of *R. chrysanthum*.

The UV-B radiation and exogenous ABA method was grounded in earlier research with appropriate adjustments [58,59]. The experiment was divided into three groups, each of which was subjected to three biological replications (*n* = 3). *R. chrysanthum* samples from M and N groups were grown in 1/4 MS medium. The culture of *R. chrysanthum* in 1/4 MS medium supplemented with ABA (at a concentration of 100 μmol/L) was group Q. After transplantation, all samples were incubated in a smart artificial climate chamber for one week before subsequent radiation treatments: group M (PAR treatment only), group N (UV-B radiation treatment), and group Q (UV-B radiation with exogenous ABA treatment).

Using a light meter (Tes-1339 Light Meter Pro.; TES Electrical Electronic Corp., Taipei, China) and a UV intensity meter (ST-513, SHH; Sentry Optron-ICS Corp., New Taipei City, China), the irradiance of the samples treated with UV-B was measured in accordance with the transmission function of the long-pass filter. Group M received only PAR treatment (50 µmol (photon) $m^{-2}$ $s^{-1}$) for 8 h per day for two days. For two days, groups N and Q were exposed to UV-B radiation for 8 h each day. UV-B radiation was provided by synthetic UV-B lamps in the wavelength range of 280–320 nm. To ensure effective UV-B radiation while excluding other light interferences, the 295 nm filter was mounted above groups N and Q. The 400 nm filter was installed in group M to remove undesired radiation. At the end of the experiment, the sample leaves were frozen in liquid nitrogen for subsequent multi-omics testing (Supplementary Figure S4).

### 4.2. Widely Targeted Metabolomics

Widely targeted metabolomics analysis of this experiment was performed by Metware Biotech Inc. (Wuhan, China). Experimental procedures and conditions closely followed the descriptions of previous studies [60]. Briefly, Analyst 1.6.3 software and Multial Quant 3.0.2 software were used to qualify and quantify the mass spectrometry data and to perform chromatographic peak integrals. Based on the Metware database (MWDB), the characterization of substances was performed based on the primary and secondary spectral data detected by mass spectrometry. Metabolite quantification was accomplished by MRM analysis using triple quadrupole mass spectrometry, during which the mass spectra of the same metabolite in different samples were integrally corrected for outgoing peaks to obtain metabolite-related data metrics.

Differential metabolites (DMs) were screened based on VIP > 1 and FC ≥ 1.5/FC ≤ 0.67. These DMs were then tagged in the KEGG database for researching the phenylpropanoid biosynthesis pathway.

### 4.3. Transcriptomics Testing

The transcriptomics assays in this work were performed by BGI Genomics Co., Ltd., Shenzhen, China. The analytical approaches and settings of the experiments strictly followed the detailed descriptions of previous studies [61].

The total RNA extraction of the samples was carried out by the CTAB method. Single-stranded circular DNA libraries were subsequently constructed and analyzed by RNA-seq.

The MGISEQ-2000 platform was used to perform RNA-seq experiments. Raw sequencing data were initially counted and filtered by SOAPnuke software (v1.4.0) to remove low-quality sequences. Subsequently, further sequence trimming was performed using trimmomatic to ensure the purity of the data. Clean reads were aligned to reference gene sequences by Bowtie2 software (v2.2.5), and the expression levels of genes and transcripts were estimated using RSEM (v1.2.8). To determine the differences in gene expression after the experimental treatments, the FPKM method was used to determine the gene expression of each transcript. The Deseq2 method based on the negative binomial distribution principle was used to explore the differential genes of *R. chrysanthum* after the corresponding experimental treatments. Differentially expressed genes were screened using FC > 1 and q-value (adjusted *p*-value) < 0.05 as standard screening criteria.

Functional annotation and classification of Unigenes used public database resources, such as KEGG, Pfam, and Swissprot. Annotation and classification of information was achieved by comparing Unigene sequences with sequences in these databases through BLAST comparison.

In this experiment, a total of nine cDNA libraries of *R. chrysanthum* samples were created, and 76.65 GB of data were acquired, with 47,598 Unigenes obtained after assembly and de-redundancy. The size of raw reads obtained was 43.69–45.44 million. After data cleaning and quality checking, the size of clean reads was 42.34–43.46 million. Q20 was more than or equal to 97.83%, whereas Q30 was greater than or equal to 92.4% (Supplementary Table S2). Unigenes had N50, N70, and N90 of not less than 1675, 1106, and 431, respectively, and a GC content of not less than 43.63% (Supplementary Table S3).

### 4.4. Quantitative Proteomic Detection of Acetylation Modifications

The quantitative proteomics assay for acetylation modifications in this study was carried out by Jingjie PTM BioLab Co. (Hangzhou, China). The assay procedure and experimental conditions were carried out in accordance with the previous experimental description [14]. A brief description is given next.

*R. chrysanthum* leaves that had been exposed to the relevant experimental treatments were subjected to protein extraction, and the levels of protein were calculated using the two-dimensional quantitative analytical kit. This was followed by trypsinization, a process that desalted the peptides using a Strata X SPE column. The treated peptides were then affinity-enriched, and the resulting peptides were desalted for LCMS/MS analysis using C18 ZipTips according to the manufacturer’s instructions. During LCMS/MS analysis, peptides were processed through a capillary source and analyzed by mass spectrometry using timsTOF Pro. The obtained mass spectrometry data were then further analyzed and annotated in the Kyoto Encyclopedia of Genes and Genomes (KEGG) database using the MaxQuant search engine (v.1.6.6.0). The FDR adjustment was set to <1%, and the differential protein screening criteria were set to *p* < 0.05; fold change (FC) ≥ 1.5.

A total of 807 differentially expressed proteins (450 with increased abundance and 357 with decreased abundance) were obtained after UV-B radiation exposure according to the differential protein screening criteria (Supplementary Figure S5A). Of the 685 acetylated differentially expressed proteins after UV-B radiation exposure, 95 increased in abundance and 590 decreased in abundance and of the 945 acetylation sites, 104 were up-regulated and 841 were down-regulated) (Supplementary Figure S5B,C).

Homology modeling of acetylated proteins followed the previous method, using NCBI BLAST to search for homologous sequences [14]. The 3D structural model of the acetylated protein was then created using the comparative protein modeling server SWISS-MODEL.

### 4.5. Determination of the OJIP Curve

With reference to the previous method [62], the dark adaption period lasted 30 min, and the fast chlorophyll fluorescence induction kinetic curve (O-J-I-P fluorescence induction curve) was measured by a Handy-PEA continuous excitation fluorometer (Hansatech, UK), with a saturated pulsed light intensity of 3000 μmol·m^−2^ s^−1^ and a measurement time of 1 s. The relevant parameters listed later were determined using JIP-test analysis, as described previously [63].

*Ψ*_o_ (probability that a trapped exciton moves an electron into the electron) = 1 − *V*_j_ ((*V*_j_ (relative variable fluorescence intensity at the J-step) = (F_j_ − F_o_)/(F_m_ − F_o_))

φ*E*_o_ (quantum yield at t = Fo for electron transport) = [1 − (F_o_/F_m_)] × *Ψ_o_*

φ*D*_o_ (quantum yield at t = Fo for energy dissipation) = 1 − F_v_/F_m_ = 1 − (F_m_ − F_o_)/F_m_

S_m_ (normalized total complementary area) = (area)/(F_m_ − F_o_)

PI_ABS_ (performance index on absorption basis) = RC/ABS [φpo/(1 − φpo)] [*Ψ_o_*/(1 − *Ψ_o_*)]

Dio/RC (dissipated energy flux per RC at t = Fo) = ABS/RC − Tro/RC

### 4.6. Determination of Chlorophyll Fluorescence Parameters

The assay was again referenced to the previous experimental procedure [64]. Determination of PSII chlorophyll fluorescence parameters and calculation of related parameters in leaves of *R. chrysanthum* were performed using the IMAGING-PAM chlorophyll fluorescence imaging system (Heinz Walz, Effeltrich, Germany). The test was carried out in a dark environment, where the temperature of the test environment was maintained at the temperature at which the test material was incubated (16 °C–18 °C).

Leaves were dark-adapted for 30 min before testing, light-induced production of Fo (minimal fluorescence) was measured, and excitation with saturating pulses of light produced maximum fluorescence Fm (maximum fluorescence). When fluorescence was reduced to Fo (the minimal fluorescence), the kinetics of fluorescence was induced by using photochemistry (1600 μmol·m^−2^·s^−1^), and saturating pulses were turned on every 20 s for the time when Fm′ (maximum fluorescence in the light) and F (actual fluorescence intensity) at any time were determined.

The final parameters measured included Fv/Fm (maximum photochemical efficiency of PSII), Y(II) (photochemical yield of PSII), Fv′/Fo′ (variable fluorescence ratio in the light), NPQ (non-photochemical quenching), and ETR (actual electron transport rate).

Fv′/Fo′= (Fm′ − Fo′)/Fo′.

### 4.7. Statistical Analysis

Orthogonal partial least squares discriminant analysis (OPLS-DA) was generated using the R package (1.0.1) MetaboAnalystR after the raw data were log2-transformed and then centered. Hierarchical cluster analysis (HCA) results for samples and metabolites are presented as heatmaps, generated by the R package ComplexHeatmap (2.9.4). The identified metabolites were annotated using the KEGG Compound Database (http://www.kegg.jp/kegg/compound/ (accessed on 6 May 2024)), and then the annotated metabolites were mapped to the KEGG pathway database. Statistical analyses were performed using IBM SPSS Statistics 26, and significant differences are indicated by different letters (*p* < 0.05). The data were analyzed using one-way analysis of variance (ANOVA). Each data set involved three biological replicates (*n* = 3). The threshold of 5% was selected to indicate substantial correlation, and the Pearson correlation test was performed.

## 5. Conclusions

**Figure 8:**
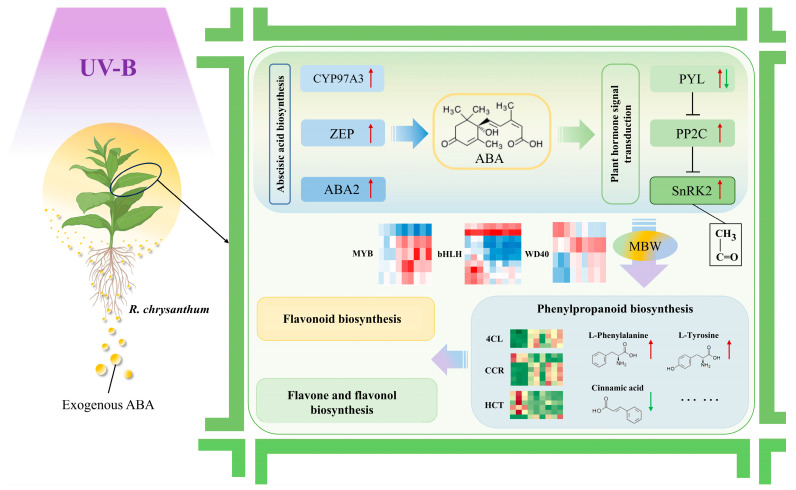
Molecular mechanisms of ABA regulation of the phenylpropanoid biosynthesis pathway in *R. chrysanthum* under UV-B stress.

The research clarifies the crucial function of abscisic acid (ABA) in regulating *R. chrysanthum*’s adaptive reaction to UV-B stress. Our comprehensive multi-omics study shows that exogenous ABA can have important regulatory effects on the phenylpropanoid biosynthesis pathway, aiding the plant in adapting to the negative impacts of increased UV-B radiation (Figure 8).

Exogenous ABA treatment was able to mitigate the decrease in photosynthetic efficiency due to UV-B radiation. In addition, exogenous ABA treatment activated the phenylpropanoid biosynthesis pathway in *R. chrysanthum* and altered the content of several flavonoids that play a role in the antioxidant defense mechanisms of the plant.

Taken together, the study reveals the complex regulatory relationship between *R. chrysanthum*’s ABA biosynthesis, the ABA signaling pathway, and the phenylpropanoid biosynthesis pathway in response to UV-B stress. UV-B radiation resulted in the differential expression of genes related to key enzymes in ABA biosynthesis (CYP97A3, ZEP, and ABA2). In addition, SnRK2, a key component of the ABA signaling cascade reaction, underwent acetylation modification after UV-B radiation exposure, which had an effect on itself.

## Figures and Tables

**Table 1:** Information about MBW-related DEGs.

Gene Annotation	Gene ID	M (FPKM)	N (FPKM)	Q (FPKM)	Type (MvsN)	Type (NvsQ)
MYB	TRINITY_DN2213_c0_g1_i4-A1	10.88	5.71	4.57	DOWN	DOWN
	TRINITY_DN236_c0_g1_i2-A1	13.27	23.93	16.35	UP	DOWN
	TRINITY_DN236_c0_g1_i3-A1	14.03	36.31	21.96	UP	DOWN
	TRINITY_DN2967_c1_g1_i1-A1	11.42	20.69	40.91	UP	UP
	TRINITY_DN4160_c0_g1_i4-A1	12.90	26.01	31.85	UP	UP
bHLH	TRINITY_DN20110_c0_g1_i1-A1	76.00	112.60	87.31	UP	DOWN
	TRINITY_DN20522_c0_g1_i1-A1	13.75	1.39	1.01	DOWN	DOWN
	TRINITY_DN27405_c0_g1_i1-A1	62.73	7.33	11.73	DOWN	UP
	TRINITY_DN4449_c0_g1_i5-A1	17.04	32.66	39.16	UP	UP
	TRINITY_DN4450_c0_g1_i2-A1	19.06	7.08	4.35	DOWN	DOWN
	TRINITY_DN4735_c0_g3_i1-A1	33.49	12.73	12.31	DOWN	DOWN
	TRINITY_DN633_c0_g3_i1-A1	36.53	12.17	9.00	DOWN	DOWN
	TRINITY_DN6387_c0_g1_i2-A1	16.49	7.62	6.37	DOWN	DOWN
	TRINITY_DN835_c0_g1_i1-A1	43.89	15.00	11.09	DOWN	DOWN
	TRINITY_DN836_c0_g3_i2-A1	23.60	15.07	13.38	DOWN	DOWN
WD40	TRINITY_DN11254_c0_g1_i1-A1	17.24	28.36	29.08	UP	UP
	TRINITY_DN21547_c0_g1_i1-A1	29.21	15.10	11.10	DOWN	DOWN
	TRINITY_DN25589_c0_g1_i1-A1	7.41	14.59	18.75	UP	UP
	TRINITY_DN6689_c0_g1_i2-A1	8.09	14.91	19.52	UP	UP

**Table 2:** Information about DEGs related to the ABA biosynthesis and signal transduction pathway.

Abbreviations for Enzymes	Gene ID	M (FPKM)	N (FPKM)	Type
CYP97A3	TRINITY_DN283_c2_g1_i6-A1	26.24	40.18	UP
ZEP	TRINITY_DN23876_c0_g1_i1-A1	3.04	10.17	UP
ABA2	TRINITY_DN4090_c0_g1_i1-A1	37.36	176.22	UP
PYL	TRINITY_DN3386_c0_g3_i3-A1	15.57	9.31	DOWN
	TRINITY_DN6964_c0_g1_i1-A1	5.73	517.12	UP
PP2C	TRINITY_DN26715_c0_g1_i1-A1	11.47	23.36	UP

## Data Availability

The data used in this study are available from the corresponding author upon submission of a reasonable request.

## Supplementary Materials

The supporting information can be downloaded at https://www.mdpi.com/article/10.3390/plants14010101/s1, Figure S1: Effect of UV-B radiation on OJIP curves and its parameters in leaves of *R. chrysanthum*; Figure S2: KEGG enrichment analysis of DEGs from R. chrysanthum under UV-B radiation and exogenous ABA treatment; Figure S3: Correlation analysis network diagram of MBW complex, DMs and DEGs; Figure S4: Flowchart of the culture of experimental material; Figure S5: Statistics on the number of DEPs in R. chrysanthum under UV-B stress; Table S1: Information about hydrophobic cluster of SnRK; Table S2: Quality statistics for Clean Reads; Table S3: Quality Indicators for Unigene. Reference [65] is cited in the Supplementary Materials.

## Abbreviations

4CL	4-Coumarate-CoA ligase
ABA	Abscisic acid
ABA2	XCanthoxin dehydrogenase
ANOVA	Analysis of variances
C12RT1	Flavanone 7-O-glucoside 2″-O-beta-L-rhamnosyltransferase
CCR	Cinnamoyl-CoA reductase
CYP97A3	Beta-ring hydroxylase
DEGs	Differentially expressed genes
DEPs	Differentially expressed proteins
DFR	Dihydroflavonol 4-reductase
DFs	Different flavonoids
DIo/RC	Dissipated energy flux per RC at t = Fo
DMS	Differential metabolites
ETR	Actual electron transport rate
FC	Fold change
Fm	Fluorescence maximum
Fm′	Maximum fluorescence in the light
Fo	Minimal fluorescence
Fo′	Minimum fluorescence in the light
FPKM	Transcript fragments per kilobase per million mapped reads
Fv′/Fo′	Variable fluorescence ratio in the light
GO	Gene Ontology
HCT	Shikimate O-hydroxycinnamoyltransferase
KEGG	Kyoto Encyclopedia of Genes and Genomes
MWDB	Metware database
NPQ	Non-photochemical quenching
OPLS-DA	Orthogonal partial least squares discriminant analysis
PAR	Photosynthetic active radiation
PI_ABS_	Performance index on absorption basis
PP2C	Serine/threonine-protein phosphatase 2A catalytic subunit
PQ	Plastoquinone
PSII	Photosystem II
PYL	Abscisic acid receptor PYR/PYL family
QA	The primary quinone acceptor
QB	The secondary quinone acceptor
Q-value	Adjusted *p* value
*R. chrysanthum*	*Rhododendron chrysanthum* Pall.
S_m_	Normalized total complementary area
SnRK2	Serine/threonine-protein kinase SRK2
TFs	Transcription factors
TRo	Trapped energy flux per reaction center
UPLC–MS/MS	Ultra-performance liquid chromatography tandem mass spectrometry
UV-B	Ultraviolet radiation b
VIP	Variable important in projection
ZEP	Zeaxanthin epoxidase
φ*D*_o_	Quantum yield at t = Fo for energy dissipation
φ*E*_o_	Quantum yield at t = Fo for electron transport
*Ψ*_o_	Probability that a trapped exciton moves an electron into the electron

## References

[1] Vanhaelewyn L., Prinsen E., Van Der Straeten D., Vandenbussche F. (2016). Hormone-controlled UV-B responses in plants. J. Exp. Bot..

[2] Shi S., Shi R., Li T., Zhou D. (2022). UV-B Radiation Effects on the Alpine Plant Kobresia humilis in a Qinghai-Tibet Alpine Meadow. Plants.

[3] Eller C.B., Barros F.d.V., Bittencourt P.R.L., Rowland L., Mencuccini M., Oliveira R.S. (2018). Xylem hydraulic safety and construction costs determine tropical tree growth. Plant Cell Environ..

[4] Parihar P., Singh S., Singh R., Singh V.P., Prasad S.M. (2015). Changing scenario in plant UV-B research:UV-B from a generic stressor to a specific regulator. J. Photochem. Photobiol. B.

[5] Falara V., Amarasinghe R., Poldy J., Pichersky E., Barrow R.A., Peakall R. (2013). The production of a key floral volatile is dependent on UV light in a sexually deceptive orchid. Ann. Bot..

[6] Zhang F., Huang J., Guo H., Yang C., Li Y., Shen S., Zhan C., Qu L., Liu X., Wang S. (2022). OsRLCK160 contributes to flavonoid accumulation and UV-B tolerance by regulating OsbZIP48 in rice. Sci. China Life Sci..

[7] Kottuparambil S., Shin W., Brown M.T., Han T. (2012). UV-B affects photosynthesis, ROS production and motility of the freshwater flagellate, Euglena agilis Carter. Aquat. Toxicol..

[8] Nocchi N., Duarte H.M., Pereira R.C., Konno T.U.P., Soares A.R. (2020). Effects of UV-B radiation on secondary metabolite production, antioxidant activity, photosynthesis and herbivory interactions in *Nymphoides humboldtiana* (Menyanthaceae). J. Photochem. Photobiol. B.

[9] Liu L.-x., Xu S.-M., Woo K.C. (2005). Solar UV-B radiation on growth, photosynthesis and the xanthophyll cycle in tropical acacias and eucalyptus. Environ. Exp. Bot..

[10] Liu H., Cao X., Azam M., Wang C., Liu C., Qiao Y., Zhang B. (2022). Metabolism of Carotenoids and β-Ionone Are Mediated by Carotenogenic Genes and PpCCD4 Under Ultraviolet B Irradiation and During Fruit Ripening. Front. Plant Sci..

[11] Liu Y., Liu J., Abozeid A., Wu K.X., Guo X.R., Mu L.Q., Tang Z.H. (2020). UV-B Radiation Largely Promoted the Transformation of Primary Metabolites to Phenols in Astragalus mongholicus Seedlings. Biomolecules.

[12] Zhou X., Chen S., Wu H., Yang Y., Xu H. (2017). Biochemical and proteomics analyses of antioxidant enzymes reveal the potential stress tolerance in *Rhododendron chrysanthum* Pall. Biol. Direct.

[13] Sun Q., Liu M., Cao K., Xu H., Zhou X. (2022). UV-B Irradiation to Amino Acids and Carbohydrate Metabolism in *Rhododendron chrysanthum* Leaves by Coupling Deep Transcriptome and Metabolome Analysis. Plants.

[14] Liu M., Sun Q., Cao K., Xu H., Zhou X. (2023). Acetylated Proteomics of UV-B Stress-Responsive in Photosystem II of *Rhododendron chrysanthum*. Cells.

[15] Pawlak-Sprada S., Arasimowicz-Jelonek M., Podgórska M., Deckert J. (2011). Activation of phenylpropanoid pathway in legume plants exposed to heavy metals. Part I. Effects of cadmium and lead on phenylalanine ammonia-lyase gene expression, enzyme activity and lignin content. Acta Biochim. Pol..

[16] Mhiri R., Koubaa I., Chawech R., Auberon F., Allouche N., Michel T. (2020). New Isoflavones with Antioxidant Activity Isolated from Cornulaca monacantha. Chem. Biodivers..

[17] Ma L., He J., Liu H., Zhou h.-h. (2018). The phenylpropanoid pathway affects apple fruit resistance to *Botrytis cinerea*. J. Phytopathol..

[18] Treutter D. (2006). Significance of flavonoids in plant resistance: A review. Environ. Chem. Lett..

[19] Shen Y., Sun T., Pan Q., Anupol N., Chen H., Shi J., Liu F., Deqiang D., Wang C., Zhao J. (2019). RrMYB5- and RrMYB10-regulated flavonoid biosynthesis plays a pivotal role in feedback loop responding to wounding and oxidation in *Rosa rugosa*. Plant Biotechnol. J..

[20] Hewage K.A.H., Yang J.F., Wang D., Hao G.F., Yang G.F., Zhu J.K. (2020). Chemical Manipulation of Abscisic Acid Signaling: A New Approach to Abiotic and Biotic Stress Management in Agriculture. Adv. Sci..

[21] Luo M., Liu J.H., Mohapatra S., Hill R.D., Mohapatra S.S. (1992). Characterization of a gene family encoding abscisic acid- and environmental stress-inducible proteins of alfalfa. J. Biol. Chem..

[22] Hartung W., Schraut D., Jiang F. (2005). Physiology of abscisic acid (ABA) in roots under stress—A review of the relationship between root ABA and radial water and ABA flows. Crop Pasture Sci..

[23] You Y., Wang Y., Zhang S., Sun X., Liu H., Guo E.Y., Du S. (2022). Different pathways for exogenous ABA-mediated down-regulation of cadmium accumulation in plants under different iron supplies. J. Hazard. Mater..

[24] Pan W., You Y., Shentu J.L., Weng Y.N., Wang S.T., Xu Q.R., Liu H.J., Du S.T. (2020). Abscisic acid (ABA)-importing transporter 1 (AIT1) contributes to the inhibition of Cd accumulation via exogenous ABA application in Arabidopsis. J. Hazard. Mater..

[25] Zhu Y., You Y., Zheng S., Li J., Wang Y., Wu R., Fang Z., Liu H., Du S. (2024). ABA-importing transporter (AIT1) synergies enhances exogenous ABA minimize heavy metals accumulations in Arabidopsis. J. Hazard. Mater..

[26] Zhang C., Ma C., Zhu L., Yao M. (2023). Simultaneous determination of protoporphyrin IX and magnesium protoporphyrin IX in *Arabidopsis thaliana* and *Camellia sinensis* using UPLC-MS/MS. Plant Methods.

[27] Balcke G.U., Handrick V., Bergau N., Fichtner M., Henning A., Stellmach H., Tissier A., Hause B., Frolov A. (2012). An UPLC-MS/MS method for highly sensitive high-throughput analysis of phytohormones in plant tissues. Plant Methods.

[28] Jiang Z., Zhou X., Li R., Michal J.J., Zhang S., Dodson M.V., Zhang Z., Harland R.M. (2015). Whole transcriptome analysis with sequencing: Methods, challenges and potential solutions. Cell Mol. Life Sci..

[29] Averbeck N.B., Durante M. (2011). Protein acetylation within the cellular response to radiation. J. Cell Physiol..

[30] Guan K.L., Xiong Y. (2011). Regulation of intermediary metabolism by protein acetylation. Trends Biochem. Sci..

[31] Choudhary C., Kumar C., Gnad F., Nielsen M.L., Rehman M., Walther T.C., Olsen J.V., Mann M. (2009). Lysine acetylation targets protein complexes and co-regulates major cellular functions. Science.

[32] Takshak S., Agrawal S.B. (2019). Defense potential of secondary metabolites in medicinal plants under UV-B stress. J. Photochem. Photobiol. B.

[33] Suzuki N., Rivero R.M., Shulaev V., Blumwald E., Mittler R. (2014). Abiotic and biotic stress combinations. New Phytol..

[34] Escobar-Bravo R., Klinkhamer P.G., Leiss K.A. (2017). Interactive Effects of UV-B Light with Abiotic Factors on Plant Growth and Chemistry, and Their Consequences for Defense against Arthropod Herbivores. Front. Plant Sci..

[35] Lee J.H., Shibata S., Goto E. (2021). Time-Course of Changes in Photosynthesis and Secondary Metabolites in Canola (*Brassica napus*) Under Different UV-B Irradiation Levels in a Plant Factory With Artificial Light. Front. Plant Sci..

[36] Armbruster U., Correa Galvis V., Kunz H.H., Strand D.D. (2017). The regulation of the chloroplast proton motive force plays a key role for photosynthesis in fluctuating light. Curr. Opin. Plant Biol..

[37] Laible P.D., Zipfel W., Owens T.G. (1994). Excited state dynamics in chlorophyll-based antennae: The role of transfer equilibrium. Biophys. J..

[38] Tóth S.Z., Schansker G., Strasser R.J. (2007). A non-invasive assay of the plastoquinone pool redox state based on the OJIP-transient. Photosynth. Res..

[39] Appenroth K.J., Stöckel J., Srivastava A., Strasser R.J. (2001). Multiple effects of chromate on the photosynthetic apparatus of Spirodela polyrhiza as probed by OJIP chlorophyll a fluorescence measurements. Environ. Pollut..

[40] Jin X., Yang G., Tan C., Zhao C. (2015). Effects of nitrogen stress on the photosynthetic CO_2_ assimilation, chlorophyll fluorescence, and sugar-nitrogen ratio in corn. Sci. Rep..

[41] Fritsche E., Schäfer C., Calles C., Bernsmann T., Bernshausen T., Wurm M., Hübenthal U., Cline J.E., Hajimiragha H., Schroeder P. (2007). Lightening up the UV response by identification of the arylhydrocarbon receptor as a cytoplasmatic target for ultraviolet B radiation. Proc. Natl. Acad. Sci. USA.

[42] Yu H., Zhang Y., Zhang Z., Zhang J., Wei Y., Jia X., Wang X., Ma X. (2020). Towards identification of molecular mechanism in which the overexpression of wheat cytosolic and plastid glutamine synthetases in tobacco enhanced drought tolerance. Plant Physiol. Biochem..

[43] Gou T., Yang L., Hu W., Chen X., Zhu Y., Guo J., Gong H. (2020). Silicon improves the growth of cucumber under excess nitrate stress by enhancing nitrogen assimilation and chlorophyll synthesis. Plant Physiol. Biochem..

[44] Tong Y., Yi S.C., Liu S.Y., Xu L., Qiu Z.X., Zeng D.Q., Tang W.W. (2022). Bruceine D may affect the phenylpropanoid biosynthesis by acting on ADTs thus inhibiting *Bidens pilosa* L. seed germination. Ecotoxicol. Environ. Saf..

[45] Muro-Villanueva F., Mao X., Chapple C. (2019). Linking phenylpropanoid metabolism, lignin deposition, and plant growth inhibition. Curr. Opin. Biotechnol..

[46] Rezayian M., Niknam V., Ebrahimzadeh H. (2018). Differential responses of phenolic compounds of *Brassica napus* under drought stress. Plant Physiol..

[47] Yan J., Wang B., Jiang Y., Cheng L., Wu T. (2014). GmFNSII-controlled soybean flavone metabolism responds to abiotic stresses and regulates plant salt tolerance. Plant Cell Physiol..

[48] Vogt T. (2010). Phenylpropanoid biosynthesis. Mol. Plant.

[49] Uhlmann A., Ebel J. (1993). Molecular cloning and expression of 4-coumarate:coenzyme A ligase, an enzyme involved in the resistance response of soybean (*Glycine max* L.) against pathogen attack. Plant Physiol..

[50] Gonzalez A., Zhao M., Leavitt J.M., Lloyd A.M. (2008). Regulation of the anthocyanin biosynthetic pathway by the TTG1/bHLH/Myb transcriptional complex in Arabidopsis seedlings. Plant J..

[51] Xu F., Ning Y., Zhang W., Liao Y., Li L., Cheng H., Cheng S. (2014). An R2R3-MYB transcription factor as a negative regulator of the flavonoid biosynthesis pathway in *Ginkgo biloba*. Funct. Integr. Genom..

[52] Hichri I., Heppel S.C., Pillet J., Léon C., Czemmel S., Delrot S., Lauvergeat V., Bogs J. (2010). The basic helix-loop-helix transcription factor MYC1 is involved in the regulation of the flavonoid biosynthesis pathway in grapevine. Mol. Plant.

[53] Petridis A., Döll S., Nichelmann L., Bilger W., Mock H.P. (2016). Arabidopsis thaliana G2-LIKE FLAVONOID REGULATOR and BRASSINOSTEROID ENHANCED EXPRESSION1 are low-temperature regulators of flavonoid accumulation. New Phytol..

[54] Luo P., Shen Y., Jin S., Huang S., Cheng X., Wang Z., Li P., Zhao J., Bao M., Ning G. (2016). Overexpression of *Rosa rugosa* anthocyanidin reductase enhances tobacco tolerance to abiotic stress through increased ROS scavenging and modulation of ABA signaling. Plant Sci..

[55] Zhu J.K. (2002). Salt and drought stress signal transduction in plants. Annu. Rev. Plant Biol..

[56] Yoshida R., Umezawa T., Mizoguchi T., Takahashi S., Takahashi F., Shinozaki K. (2006). The regulatory domain of SRK2E/OST1/SnRK2.6 interacts with ABI1 and integrates abscisic acid (ABA) and osmotic stress signals controlling stomatal closure in Arabidopsis. J. Biol. Chem..

[57] Umezawa T., Nakashima K., Miyakawa T., Kuromori T., Tanokura M., Shinozaki K., Yamaguchi-Shinozaki K. (2010). Molecular basis of the core regulatory network in ABA responses: Sensing, signaling and transport. Plant Cell Physiol..

[58] Yu W., Gong F., Xu H., Zhou X. (2024). Molecular Mechanism of Exogenous ABA to Enhance UV-B Resistance in *Rhododendron chrysanthum* Pall. by Modulating Flavonoid Accumulation. Int. J. Mol. Sci..

[59] Lyu J., Wang C., Liang D.Y., Liu L., Pandey L.K., Xu H.W., Zhou X.F. (2019). Sensitivity of wild and domesticated *Rhododendron chrysanthum* to different light regime (UVA, UVB, and PAR). Photosynthetica.

[60] Gong F., Yu W., Zeng Q., Dong J., Cao K., Xu H., Zhou X. (2023). *Rhododendron chrysanthum*’s Primary Metabolites Are Converted to Phenolics More Quickly When Exposed to UV-B Radiation. Biomolecules.

[61] Yu W., Gong F., Zhou X., Xu H., Lyu J., Zhou X. (2024). Comparative Metabolomics and Transcriptome Studies of Two Forms of *Rhododendron chrysanthum* Pall. under UV-B Stress. Biology.

[62] Liu M., Lin X., Cao K., Yang L., Xu H., Zhou X. (2023). Multi-Omic Analysis Reveals the Molecular Mechanism of UV-B Stress Resistance in Acetylated RcMYB44 in *Rhododendron chrysanthum*. Genes.

[63] Strasser R.J., Tsimilli-Michael M., Qiang S., Goltsev V. (2010). Simultaneous in vivo recording of prompt and delayed fluorescence and 820-nm reflection changes during drying and after rehydration of the resurrection plant *Haberlea rhodopensis*. Biochim. Biophys. Acta.

[64] Zeng Q., Dong J., Lin X., Zhou X., Xu H. (2024). Isolation and Identification of *Acer truncatum* Endophytic Fungus Talaromyces verruculosus and Evaluation of Its Effects on Insoluble Phosphorus Absorption Capacity and Growth of Cucumber Seedlings. J. Fungi.

[65] Gong F., Yu W., Cao K., Xu H., Zhou X. (2024). Rctrp5 transcription factor mediates the molecular mechanism of ligninbiosynthesis regulation in r. Chrysanthum against uv-b stress. Int. J. Mol. Sci..

