# Supplementary material for: Multi-Omics Research Reveals the Effects of the ABA-Regulated Phenylpropanoid Biosynthesis Pathway on the UV-B Response in Rhododendron chrysanthum Pall
Source: Plants (Basel). 2025 Jan 1;14(1):101. doi: 10.3390/plants14010101 (PMC11723134; doi:10.3390/plants14010101)
Supplement: Supplementary file 1 [file plants-14-00101-s001.zip › Supplementary Figuers.pdf]

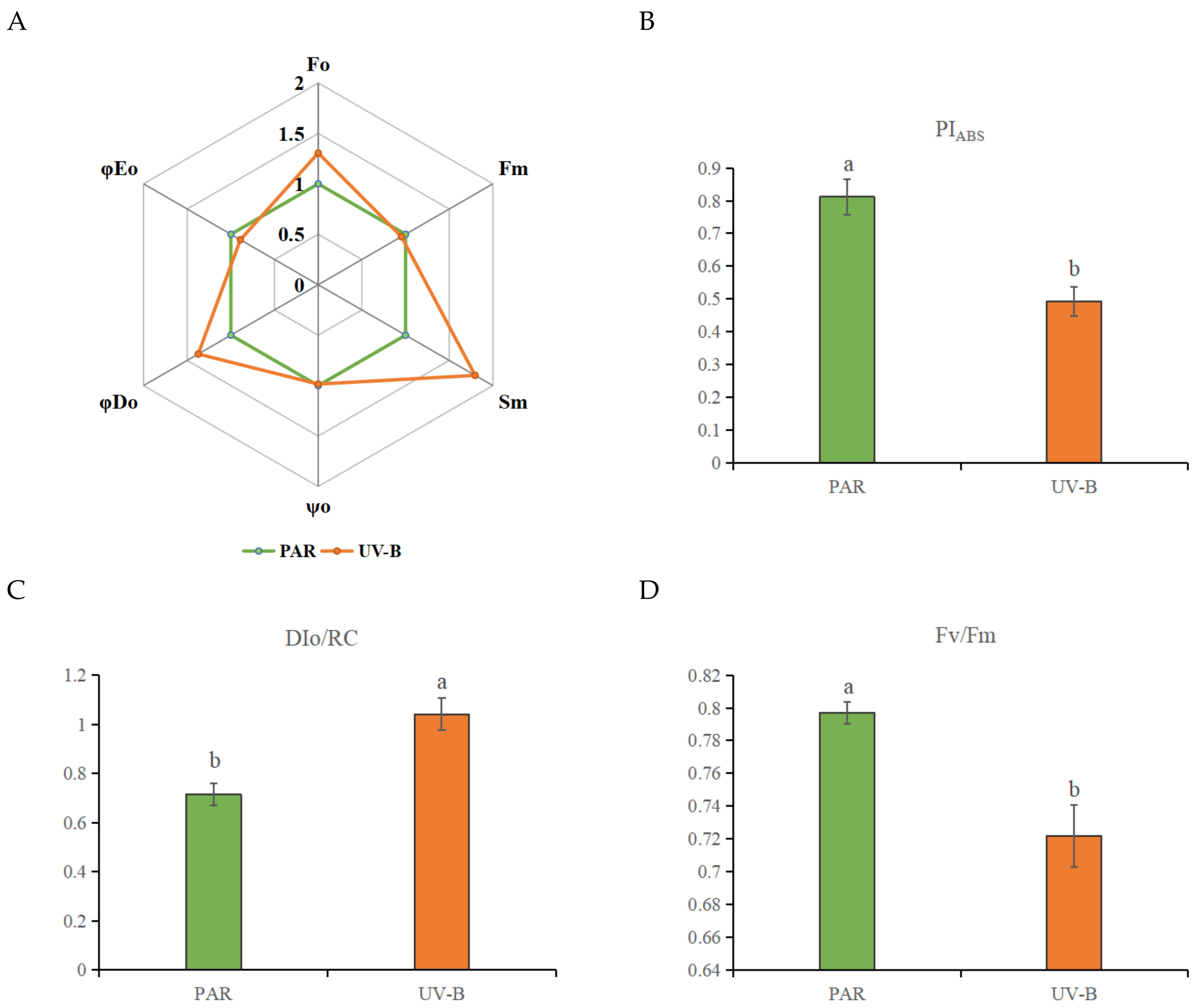

Supplementary Figure S1. Effect of UV-B radiation on OJIP curves and its parameters in leaves of *R. chrysanthum*. (A) Radar plots showing the fluctuations of each parameter under UV-B stress; (B-D) Bar graphs indicating the changes in the photosystem II-related indices, PI_{ABS}, DIo/RC, and Fv/Fm, respectively, after UV-B radiation. The three biological duplicate experiments' means are shown by the height of the bar graph ($n = 3$), and SD of the three samples is shown by the error bars. Significant changes across data groups are indicated by different letter markers ($p < 0.05$).

Some of these results are quoted from previous experiment with minor modifications[1].

1. Gong, F., W. Yu, K. Cao, H. Xu and X. Zhou. "Rctrp5 transcription factor mediates the molecular mechanism of lignin biosynthesis regulation in r. Chrysanthum against uv-b stress." *Int J Mol Sci* 25 (2024): 10.3390/ijms25179205.

A

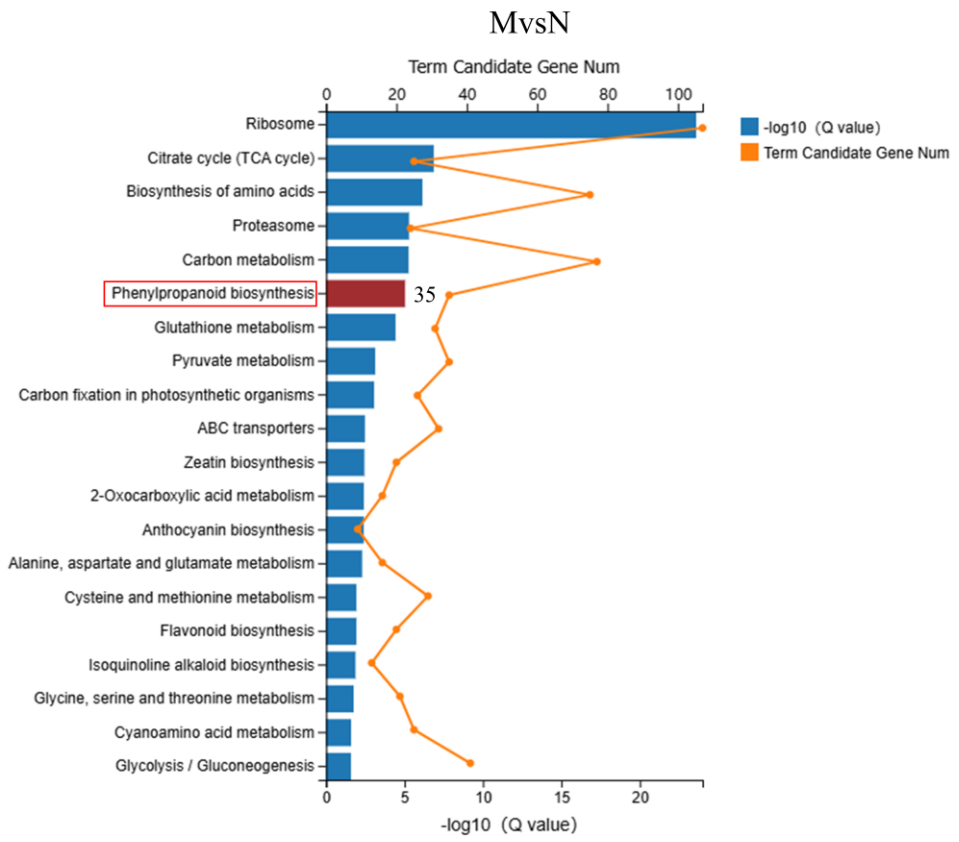

B

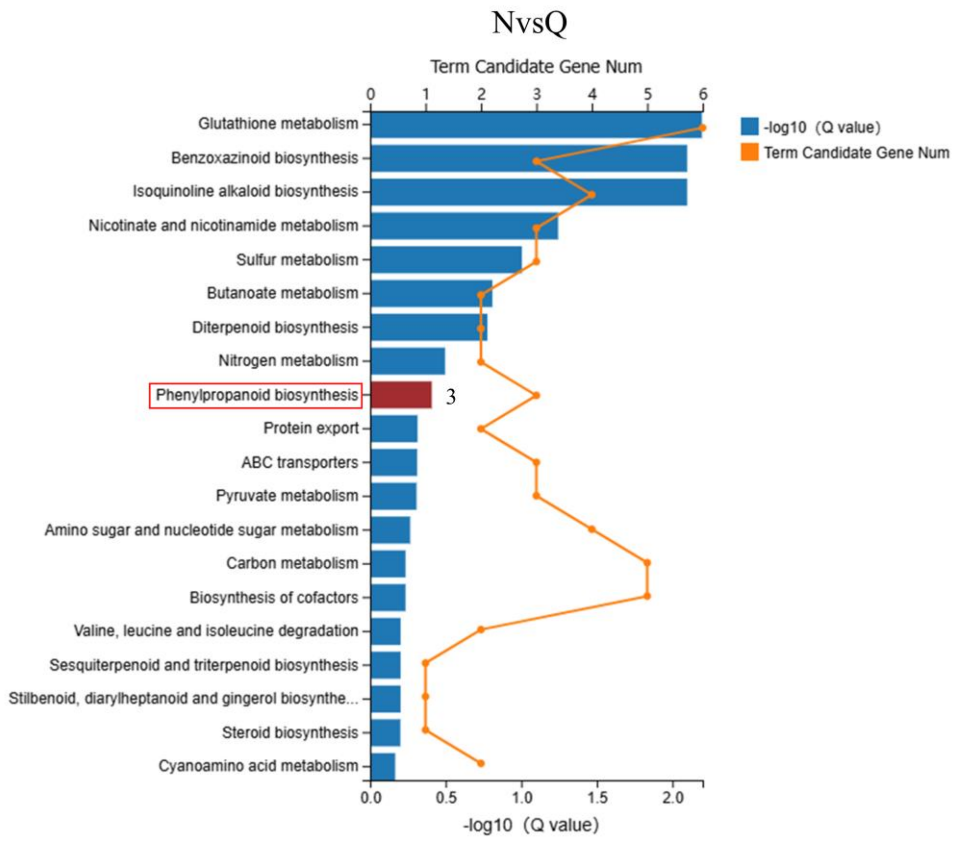

Supplementary Figure S2. KEGG enrichment analysis of DEGs from *R. chrysanthum* under UV-B radiation and exogenous ABA treatment. (A) Bar graph of KEGG enrichment analysis of DEGs of *R. chrysanthum* under UV-B radiation; (B) Bar graph of KEGG enrichment analysis of DEGs of *R. chrysanthum* treated with exogenous ABA.

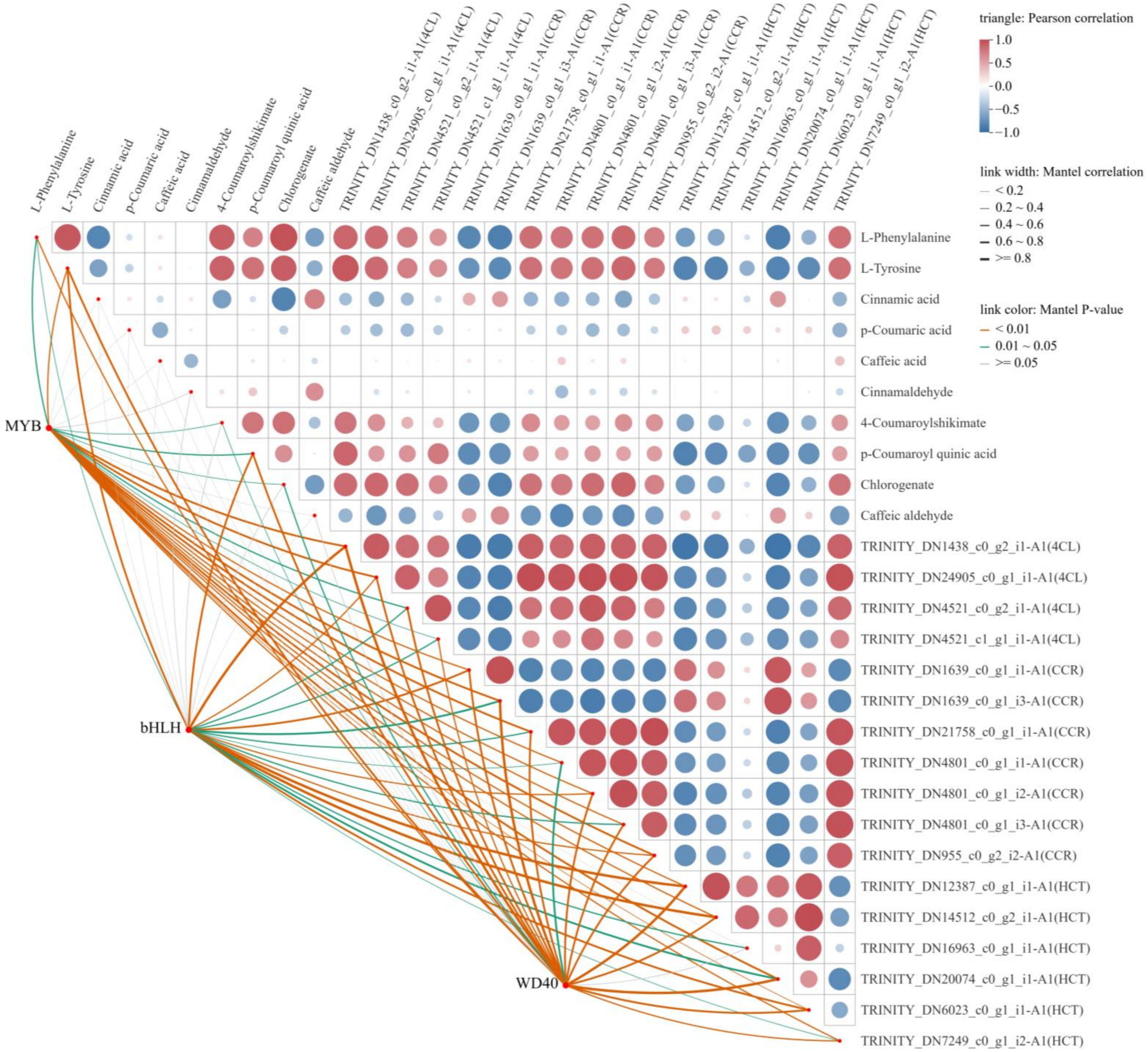

Supplementary Figure S3. Correlation analysis network diagram of MBW complex, DMs and DEGs. The upper right half is a heatmap of the correlation between DMs and DEGs, where the color and size of the circles indicate the size of the correlation coefficient, and the larger the circle the darker red the color, indicating a stronger correlation. Red color indicates positive correlation and blue color indicates negative correlation. The connecting lines in the lower left indicate the correlation of MBW complex (MYB, bHLH, and WD40) with DMs and DEGs, and the thickness and color of the connecting lines indicate the magnitude and significance of the correlation coefficients. The thicker the line, the darker the color, the stronger and more significant the corresponding correlation.

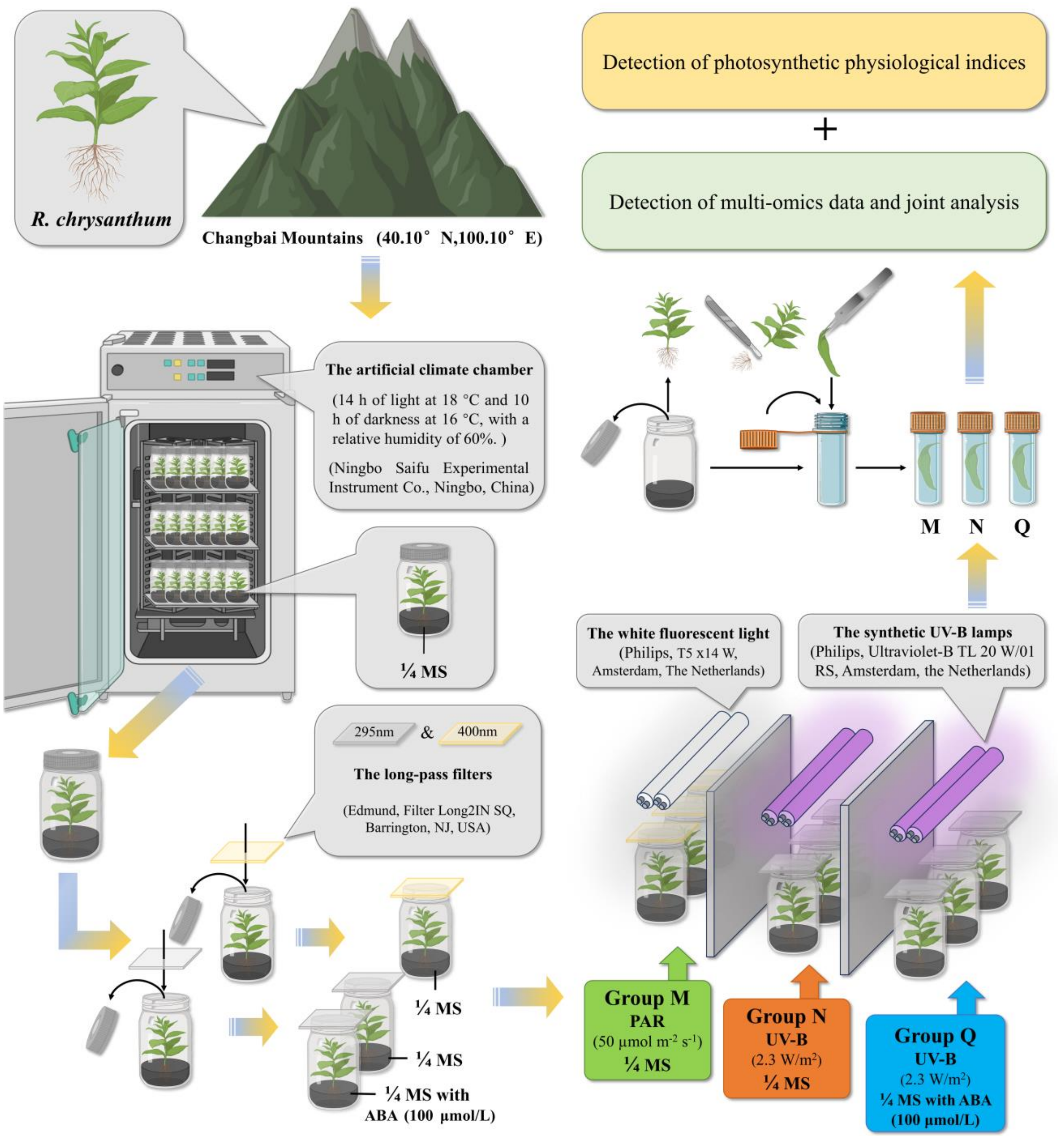

Supplementary Figure S4. Flowchart of the culture of experimental material. Blue and yellow gradient arrows point to indicate the sequence of steps in the experimental process. The grey box shows the model information of the relevant apparatus and equipment

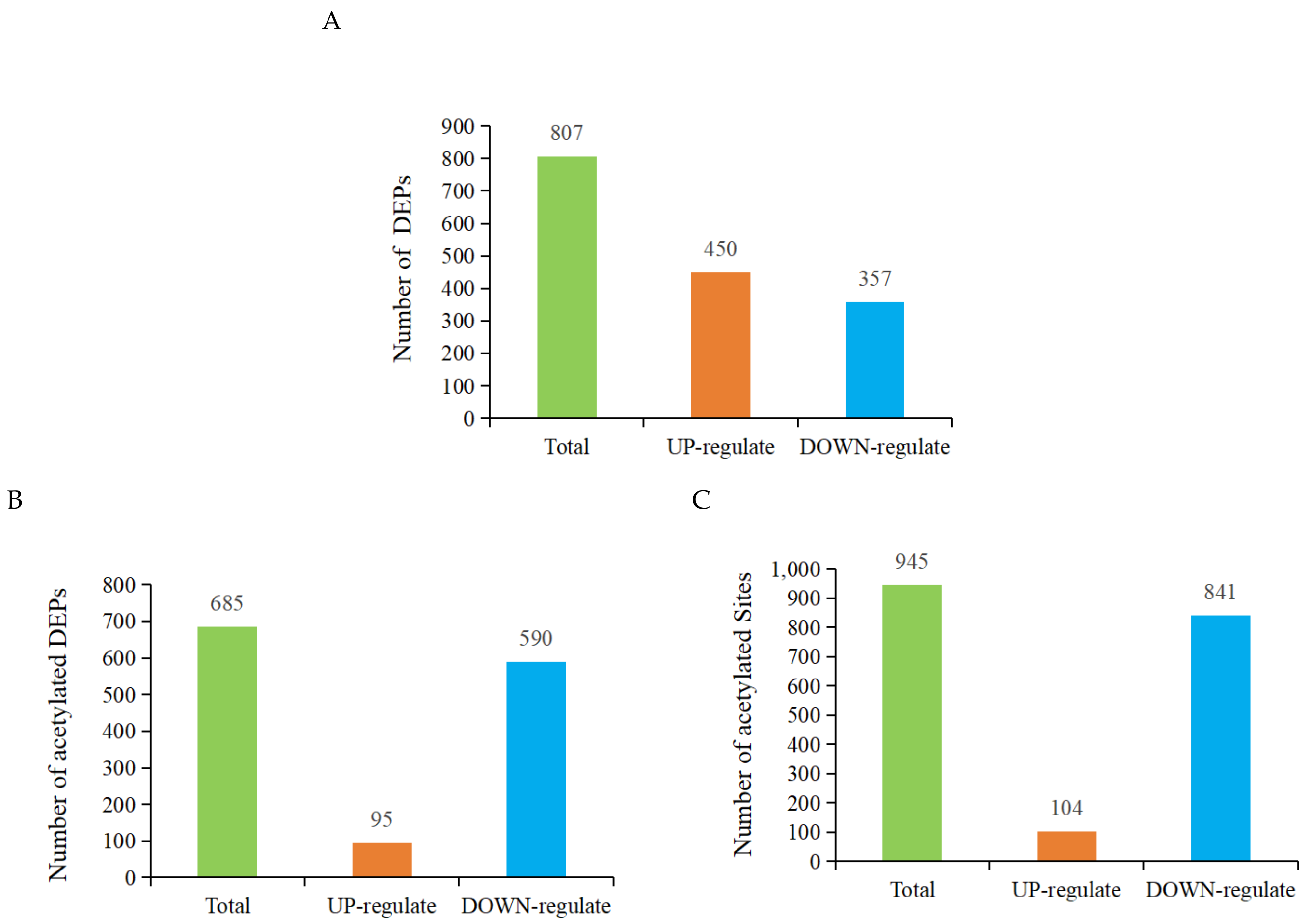

Supplementary Figure S5. Statistics on the number of DEPs in *R. chrysanthum* under UV-B stress. (A) Histogram of the number of differentially expressed proteins; (B) Histogram of the number of differentially acetylated proteins; (C) Histogram of the number of differentially acetylated protein sites.
